# Hemodialysis Access–Related High-Output Heart Failure: From Mechanisms to Multidisciplinary Management

**DOI:** 10.1016/j.jaccas.2026.107534

**Published:** 2026-04-01

**Authors:** Chukwuemezie Kamanu, Estefania Oliveros, Yevgeniy Brailovsky

**Affiliations:** a Department of Medicine, Thomas Jefferson University Hospital, Philadelphia, Pennsylvania, USA; b Division of Cardiology, Department of Medicine, Columbia University Irving Medical Center, New York, New York, USA

**Keywords:** acute heart failure, echocardiography, hemodynamics

**Figure:**
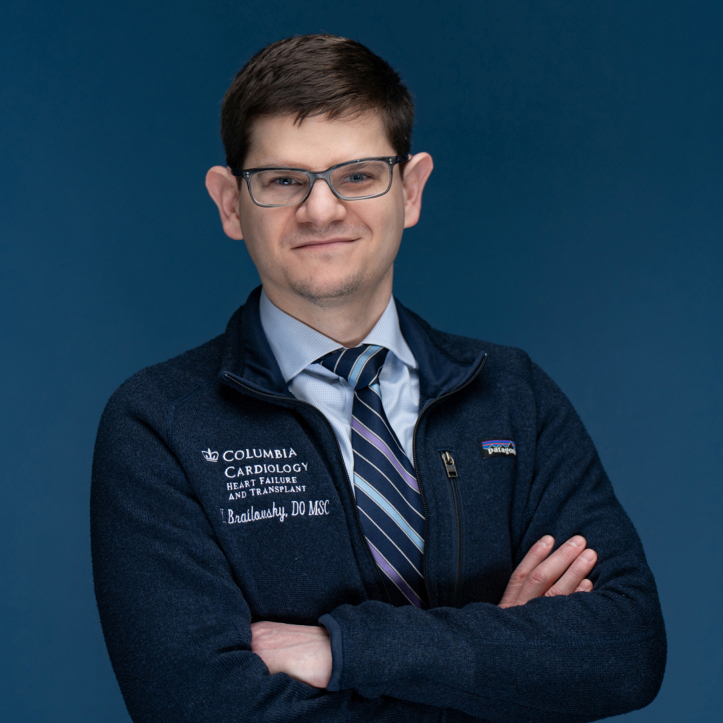

The intersectionality between heart failure and chronic kidney disease (CKD) is often seen. Both affect older adults and individuals with hypertension, diabetes, and left ventricular (LV) systolic and/or diastolic dysfunction.^1^ Heart failure affects the renal blood flow and causes tubular necrosis, whereas CKD contributes to LV remodeling, fibrosis, and LV dysfunction in the presence of fluid overload, anemia, uremia, and activation of the renin-angiotensin-aldosterone and sympathetic system. This altered physiological setting will also affect the pulmonary vascular bed causing pulmonary hypertension (PH). Seminal studies suggest that up to 42% of patients receiving dialysis develop heart failure and PH.^2^ As CKD progresses to end-stage renal disease (ESRD), the creation of a hemodialysis arteriovenous (AV) access adds hemodynamic stress that can precipitate or worsen heart failure.^3^ AV access–related high-output heart failure is serious, underdiagnosed, and most commonly occurs in patients with established cardiovascular disease or risk factors. Given the high prevalence of these conditions in the dialysis population, a substantial proportion of patients remain vulnerable.^3^

**Visual Summary:**
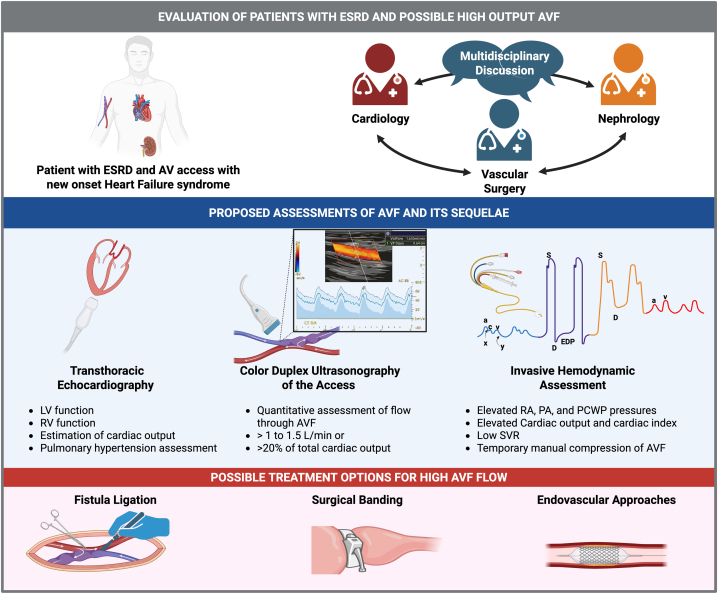
Evaluation of Patients With ESRD and Suspected High-Output Heart Failure AV = arteriovenous; AVF = arteriovenous fistula; ESRD = end-stage renal disease; LV = left ventricular; PA = pulmonary artery; PCWP = pulmonary capillary wedge pressure; RA = right atrial; RV = right ventricular; SVR = systemic vascular resistance.

Although most heart failure occurs with normal or reduced cardiac output, AV fistulas (AVFs) and grafts can lead to high-output heart failure when they are sizable. The AVF is a low resistance circuit that can cause an increase venous return to the right side of the heart. This condition is driven by a disproportionate reduction in systemic vascular resistance and increased metabolic demand, resulting in reduced afterload, enhanced stroke volume, and elevated cardiac output. The resulting increase in preload, together with neurohormonal activation secondary to renal hypoperfusion, promotes sodium and volume retention and may culminate in overt heart failure in susceptible patients.^4^

PH in the setting of ESRD is multifactorial. First, PH results from postcapillary pulmonary venous hypertension but ultimately can develop precapillary PH over time. The high-output state will result in high pulmonary pressures (from high flow through right ventricle-pulmonary artery circuit) even in the absence of intrinsic pulmonary vascular disease. The sheer stress in the pulmonary vascular bed related to long-standing increased pulmonary blood flow will cause remodeling, rising right-sided afterload and high pulmonary vascular resistance.^5^ These changes may evolve rapidly, with symptoms appearing within months. New-onset heart failure or persistent difficulty achieving euvolemia in patients with AV access should prompt evaluation for high-output heart failure or PH. The clinical implications are substantial. In a Mayo Clinic cohort of 120 patients with high-output heart failure, nearly 40% died within 3 years, highlighting the need for early recognition and timely intervention.^6^

In this issue of *JACC: Case Reports*, Son et al^7^ present a case of a patient with ESRD, who underwent AVF placement 1 year earlier, who presented with progressive dyspnea. The authors rightfully considered a broad differential of the patient's symptoms, including high-output heart failure.^7^

Evaluation of suspected AV access–related high-output heart failure focuses on quantifying cardiac output and assessing the hemodynamic response to access flow.^5^ A stepwise approach combines noninvasive imaging with invasive hemodynamic assessment. Transthoracic echocardiography and color duplex ultrasonography of the access are typically used first, with right heart catheterization performed to confirm the diagnosis when indicated. Serial echocardiography before and after temporary fistula occlusion can reveal changes in cardiac performance attributable to access flow.^8^ Duplex ultrasonography permits direct measurement of fistula flow and is useful for identifying hemodynamically significant access, which correlates with high-output heart failure and PH.^5^ Hemodynamic assessment in the catheterization laboratory may elucidate the presence of PH due to high-output or unveil underlying precapillary PH. Temporary and brief occlusion of the AVF, using manual pressure or blood pressure cuff, may show the Nicoladoni-Branham sign with a sudden drop in heart rate, a rise in blood pressure, a decrease in the stroke volume and high cadiac output, and, in the absence of precapillary PH, a reduction of the mean pulmonary pressure.^9^ However, caution must be used with this technique, as an improper occlusion of the AVF can cause thrombosis, severe pain, and arm swelling. This immediate effect has been related to the effect of blood pH on the action of choline esterase affecting the baroreceptor mediated effect via the vagus response.^9^

High-flow AVFs are commonly defined by flows >1-1.5 L/min or accounting for more than 20% of total cardiac output.^10^ Useful but somewhat arbitrary guidelines for ranges of blood flow within typical dialysis access have been described: low (600 mL/min), normal (600-1,500 mL/min), and high (1,500-4,000 mL/min).^11^ High flow results in lumen sclerosis or lumen dilation. Extreme high flows will show rapid dilation and formation of “aneurysmal” or “large AVFs.” Overfunctioning AVFs will cause issues with fluid retention and achievement of effective dialysis.^11^ Observational studies indicate that patients who develop PH have substantially higher access flows than those without pulmonary vascular disease.^10^ Nevertheless, access flow alone does not establish hemodynamic significance. High-output heart failure reflects the interaction between access demand and myocardial reserve. Patients with preserved cardiac reserve may tolerate even markedly elevated flows without developing symptoms. Demonstrating a meaningful reduction in cardiac output after fistula occlusion is therefore essential for confirming the diagnosis. Right heart catheterization remains the gold standard, showing elevated cardiac output or cardiac index in the setting of low systemic vascular resistance and clinical signs of heart failure.^6^ The hemodynamic load is related to the chronic activation the renin-angiotensin-aldosterone system and the recruitment of small increments of blood from the liver, spleen, and splanchnic circulation, which result in the hyperdynamic circulation, worsening sodium retention, an increase in stressed blood volume, and congestion. In addition, patients with a hemodynamically significant fistula will have an increased nitric oxide production, which also causes abnormal nitric oxide pathways affecting the pulmonary vascular bed and systemic vascular resistance. In a Mayo Clinic cohort, a transthoracic echocardiography–derived cardiac index of ≥3.54 L/min/m^2^ identified high-output heart failure with 62% sensitivity and 96% specificity, while a Doppler-estimated right ventricular systolic pressure of ≥42 mm Hg showed 92% sensitivity and 100% specificity.^6^

The case presented by Son et al^7^ in this issue of *JACC: Case Reports* highlights important steps for evaluation of patients with ESRD suspected of having high-output heart failure. The work-up for this patient included transthoracic echocardiogram, demonstrating several findings of impaired diastolic function, LV remodeling, and signs of elevated cardiac output. Subsequent right heart catheterization was consistent with the diagnosis of high-output heart failure with elevated filling pressures and substantially elevated cardiac output, which reduced with manual compression of the fistula. The authors also rightfully excluded alternative causes of high-output state.^7^

Management of AV access–related high-output heart failure aims to reduce or eliminate the pathologic shunt. Fistula ligation effectively reverses adverse hemodynamics but sacrifices dialysis access, limiting its suitability to patients with a functioning kidney transplant or those able to transition to peritoneal dialysis or catheter-based dialysis access. Retrospective studies and meta-analyses have demonstrated improvements in cardiac remodeling, ventricular function, and ejection fraction following ligation.^12^ Flow-reduction strategies offer alternatives that preserve access. Surgical banding, which narrows the fistula at specific points, can significantly reduce flow and improve heart failure symptoms, as shown in small cohorts including patients with markedly elevated baseline flows.^13^ Endovascular approaches such as stent-based flow reduction have also been explored, but long-term durability and efficacy are inconsistent.^14^ No head-to-head trials have compared ligation, banding, and stenting, emphasizing the need for prospective studies to define optimal patient selection, flow thresholds, and potential survival benefit.

In the absence of clear guidelines, dialysis access planning should account for baseline cardiovascular risk. Patients with preexisting heart failure, PH, or right ventricular dysfunction may benefit from alternatives to AV access, including peritoneal dialysis or tunneled dialysis catheters. A multidisciplinary approach involving nephrology, cardiology, vascular surgery, and the patient is essential to individualize access selection and management.^5^ Careful assessment of ventricular function, pulmonary pressures, and cardio-pulmonary comorbidities should guide decisions regarding fistula size, flow modulation, or alternative dialysis modalities.^5^ Given the limited evidence base, decisions regarding access modification or ligation in patients with high-output heart failure or PH should rely on comprehensive evaluation, multidisciplinary discussion, and shared decision-making.

In the case presented by Son et al,^7^ the authors highlighted the need for multidisciplinary team to weigh the risks and benefits of each treatment modality and tailor-specific approach for this patient.

Finally, biomarkers represent a promising but underdeveloped tool for anticipating high-output heart failure in patients with AV access. Traditional markers such as natriuretic peptides and troponins provide prognostic information about cardiovascular stress, but their predictive utility for high-output states is limited by renal clearance and nonspecificity for cardiac output. Emerging biomarkers linked to myocardial remodeling and adverse outcomes may offer a more targeted approach, and prospective studies are needed to determine whether they can support early risk stratification and prevention of high-output heart failure.

## Funding Support and Author Disclosures

Dr Brailovsky has received consulting/advisory board fees from Alnylam, Pfizer, BridgeBio, and AstraZeneza. All other authors have reported that they have no relationships relevant to the contents of this paper to disclose.
